# Evaluating the effectiveness of a novel somatostatin receptor 2 antagonist, ZT-01, for hypoglycemia prevention in a rodent model of type 2 diabetes

**DOI:** 10.3389/fphar.2024.1302015

**Published:** 2024-02-28

**Authors:** Ninoschka C. D’Souza, Julian A. Aiken, Emily G. Hoffman, Sara C. Atherley, Sabrina Champsi, Nadia Aleali, Dorsa Shakeri, Maya El-Zahed, Nicky Akbarian, Mehran Nejad-Mansouri, Parinaz Z. Bavani, Richard L. Liggins, Owen Chan, Michael C. Riddell

**Affiliations:** ^1^ School of Kinesiology and Health Science, York University, Toronto, ON, Canada; ^2^ Zucara Therapeutics, Vancouver, BC, Canada; ^3^ Department of Internal Medicine, Division of Endocrinology, University of Utah, Salt LakeCity, UT, United States

**Keywords:** type 2 diabetes, glucagon, hypoglycemia, counterregulation, somatostatin receptor antagonist

## Abstract

**Background:** Elevated levels of somatostatin blunt glucagon counterregulation during hypoglycemia in type 1 diabetes (T1D) and this can be improved using somatostatin receptor 2 (SSTR2) antagonists. Hypoglycemia also occurs in late-stage type 2 diabetes (T2D), particularly when insulin therapy is initiated, but the utility of SSTR2 antagonists in ameliorating hypoglycemia in this disease state is unknown. We examined the efficacy of a single-dose of SSTR2 antagonists in a rodent model of T2D.

**Methods:** High-fat fed (HFF), low dose streptozotocin (STZ, 35 mg/kg)-induced T2D and HFF only, nondiabetic (controls-no STZ) rats were treated with the SSTR2 antagonists ZT-01/PRL-2903 or vehicle (*n* = 9–11/group) 60 min before an insulin tolerance test (ITT; 2–12 U/kg insulin aspart) or an oral glucose tolerance test (OGTT; 2 g/kg glucose via oral gavage) on separate days.

**Results:** This rodent model of T2D is characterized by higher baseline glucose and HbA1c levels relative to HFF controls. T2D rats also had lower c-peptide levels at baseline and a blunted glucagon counterregulatory response to hypoglycemia when subjected to the ITT. SSTR2 antagonists increased the glucagon response and reduced incidence of hypoglycemia, which was more pronounced with ZT-01 than PRL-2903. ZT-01 treatment in the T2D rats increased glucagon levels above the control response within 60 min of dosing, and values remained elevated during the ITT (glucagon Cmax: 156 ± 50 vs. 77 ± 46 pg/mL, *p* < 0.01). Hypoglycemia incidence was attenuated with ZT-01 vs. controls (63% vs. 100%) and average time to hypoglycemia onset was also delayed (103.1 ± 24.6 vs. 66.1 ± 23.6 min, *p* < 0.05). ZT-01 administration at the OGTT onset increased the glucagon response without exacerbating hyperglycemia (2877 ± 806 vs. 2982 ± 781), potentially due to the corresponding increase in c-peptide levels (6251 ± 5463 vs. 14008 ± 5495, *p* = 0.013).

**Conclusion:** Treatment with SSTR2 antagonists increases glucagon responses in a rat model of T2D and results in less hypoglycemia exposure. Future studies are required to determine the best dosing periods for chronic SSTR2 antagonism treatment in T2D.

## 1 Introduction

The intensification in glycemic management with new diabetes-related therapies over recent decades has resulted in less patient exposure to hyperglycemia, better lipid control and less risk for a majority of long-term complications from micro- and macrovascular disease (Harding et al., 2019). However, despite new technologies and therapies that target hyperglycemia, such as new oral agents (including GLP-1 analogs), new insulin and continuous glucose monitors (CGM), hypoglycemia remains a major barrier to tight glycemic control, even in patients with type 2 diabetes (T2D) (Balijepalli et al., 2017; Goyal et al., 2017; Hædersdal et al., 2018). The frequency and extent of hypoglycemia in people living with T2D are often underappreciated, relative to hypoglycemia in type 1 diabetes (T1D), especially in individuals with long-standing T2D (Heller et al., 2020). Data from large clinical trials (e.g., ACCORD, ADVANCE and UKPDS) suggest that close to 75% of individuals living with T2D were unaware of significant and repeated episodes of level 1 and level 2 hypoglycemia exposure (interstitial glucose ≤3.9 or <3.0 mmol/L, respectively) which were detected by CGM (Freeman, 2019). Although most of the events detected in these studies occurred in subjects using insulin therapy or insulin secretagogues, a number of events also occurred in subjects on other medications (Gehlaut et al., 2015). In one large trial evaluating the incidence of hypoglycemia using self-monitoring of blood glucose and/or interstitial continuous glucose monitors, it was found that −56% of T2D patients on insulin had hypoglycemic events, as defined as a glucose ≤60 mg/dL, over a 72-h observational period (Zick et al., 2007). This incidence of hypoglycemia in this patient population only increases when combined with other comorbidities such as cardiovascular disease, congestive heart failure and chronic kidney disease (Zick et al., 2007; Yun et al., 2015). The increasing prevalence of hypoglycemia in T2D is also reflected in the higher rates of hospitalizations for episodes of severe hypoglycemia, where the individual is unable to self-treat, costing an average of ∼$10,139 per patient visit (Goyal et al., 2017; Freeman, 2019).

In the absence of diabetes, several counter regulatory hormones rise when glucose levels drop below a hemostatic setpoint (−5.0 mmol/L), including glucagon, cortisol, growth hormone and catecholamines (Reno et al., 2013). These counterregulatory hormones, along with a reduction in endogenous insulin secretion, play a critical role in limiting exposure to glycemia below −4.0 mmol/L in the healthy organism. Of these responses, glucagon secretion from pancreatic α-cells typically increases dramatically when glucose reaches ∼3.6 mmol/L, and this response plays a primary role in preventing hypoglycemia overnight, during exercise and between meals by stimulating up to 90% of hepatic glucose output, with catecholamines and other counterregulatory hormones serving as second-line defences (Rivera et al., 2010). In T1D, there is defective glucagon counterregulation to hypoglycemia soon after diagnosis (Arbelaez et al., 2014), despite normal or even elevated glucagon content found in pancreatic α-cells (Bonnet-Serrano et al., 2018). The inability to secrete glucagon normally when exposed to hypoglycemia in T1D may be because of intra-islet dysregulation in the absence of insulin secretion from pancreatic ß-cells and/or elevated somatostatin (SST) signalling from nearby pancreatic δ-cells (Vergari et al., 2019). However, it is currently unclear if the same physiologic dysregulation in SST signalling exists in T2D, but those with long-standing disease, appear to have a reduced or absent glucagon response to hypoglycemia, especially in those treated with insulin who tend to have recurrent episodes of iatrogenic hypoglycemia (Murayama et al., 1989; Dagogo-Jack et al., 1993; Cryer, 2012; Oyer, 2013).

SST receptor 2 (SSTR2), the predominant SST receptor expressed by pancreatic α-cells in humans and rodents (Blodgett et al., 2015), can be antagonized by highly selective SSTR2 antagonists to fully or partially restore glucagon responses to hypoglycemia in rodent models of T1D (Yue et al., 2012; 2013; Karimian et al., 2013; Leclair et al., 2016; Farhat et al., 2022; GhavamiNejad et al., 2022) and in non-diabetic rats who are pre-exposed to recurrent hypoglycemia to generate a model of glucagon counterregulatory failure (Hoffman et al., 2021). The pharmacologic inhibition of the SSTR2 for the intent of enhancing glucagon counterregulation has recently been reviewed by Hoffman et al., 2024. ZT-01, a highly specific and potent SSTR2 antagonist, has recently entered human clinical trials assessing its pharmacodynamic effect on glucagon counterregulation in adults living with T1D (NCT05007977: Effect of ZT-01 on Glucagon During Hypoglycemia in Type 1 Diabetes Mellitus)^1^ (Abitbol et al., 2023)^2^, as well as hypoglycemia prevention in adults living with T1D who have significant nocturnal hypoglycemia (NCT05762107: A Study of the Effect of ZT-01 on Night-time Hypoglycemia in Type 1 Diabetes [ZONE])^3^, these studies will test the hypothesis that SSTR2 antagonism has the potential to improve the glucagon counterregulatory response in T1D that is likely impaired, at least in part, because of elevated pancreatic secretion of SST.

T2D, a disease of insulin resistance and relative insulin deficiency (Kahn et al., 2014), is also characterized by elevated circulating glucagon levels in the post-meal state (Ito et al., 2021), which may contribute to meal-related dysglycemia. However, individuals with advanced or long-standing T2D may also have glucagon counterregulatory deficiency to hypoglycemia (Segel et al., 2002; Stanley et al., 2019). To evaluate the effects of SSTR antagonists in this context, we developed a high-fat fed (HFF) low dose streptozotocin (STZ)-induced rodent model of late-stage T2D, adapted from a previously existing model (Srinivasan et al., 2005) due to its similarities to the T2D clinical phenotype, to better characterize glucagon dysregulation in T2D and to assess if SSTR2 antagonists have therapeutic potential for hypoglycemia prevention in this form of diabetes.

In this study, we establish a rodent model of T2D, characterize the counterregulatory response to hypoglycemia and evaluate proof of concept and efficacy of the SSTR2 antagonists PRL-2903 and ZT-01 to prevent insulin-induced hypoglycemia. Additionally, we assessed the glycemic and plasma hormone responses to SSTR2 antagonists under basal and meal-simulated conditions using an oral glucose tolerance test (OGTT) to better understand their therapeutic potential for people living with T2D.

## 2 Methods

### 2.1 T2D induction and study protocol

**FIGURE 1:**
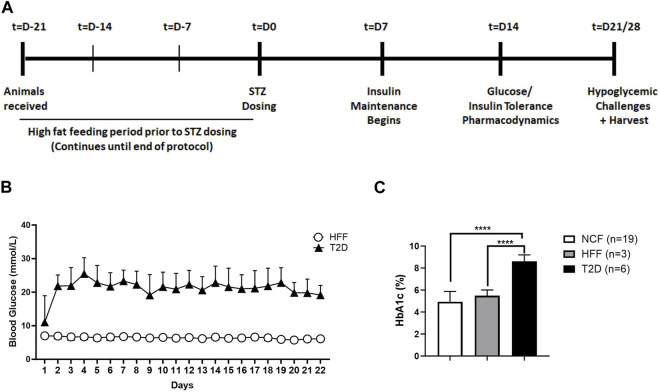
Schematic representation of study protocol and model characterization. General study protocol assessing hormone responses to drug treatment in the absence and presence of hypoglycemia. The protocol spans a period of 5–6 weeks starting with an initial high-fat feeding period prior to diabetes induction, followed by insulin maintenance and finally various tests at differing levels of glycemia, with and without SSTR2 antagonist **(A)**. Daily monitoring graph **(B)** illustrating blood glucose levels of animals with T2D and high-fat fed (HFF) controls over the duration of the study. **(C)** HbA1c levels in normal chow-fed (NCF) and HFF control animals and animals with T2D. All data are represented as mean ± SD.

Eight-week-old male Sprague Dawley rats (strain 001, Charles River Laboratories, Montreal, QC, Canada) were used in this study. Animal study procedures received ethics approval from the York University Animal Care Committee and were conducted in accordance with the Canadian Council for Animal Care guidelines (Protocol #2017-7). Rats were housed within York University’s animal care facility under a 12-h light/dark cycle, with controlled temperatures (23°C–25°C), and *ad libitum* access to food and water. Rats were fed a high-fat diet (60% fat, 20% carbohydrates, 20% protein, ResearchDiets, Burlington, Canada; Cat#: D12492) to induce baseline obesity and insulin resistance in preparation for diabetes induction using low-dose STZ. Five-week-old normal chow-fed (NCF) control animals were given a standard chow diet (Purina Lab diet 5012, St. Louis, MO, UnitedStates). The general timeline for the study protocol is summarized in Figure 1A. After 3 weeks of high-fat feeding, rats were fasted overnight (17:00 to 9:00) before receiving an intraperitoneal (IP) injection of STZ (35 mg/kg, dissolved in saline, Sigma-Aldrich, Oakville, Canada) to induce partial β cell loss and moderate hyperglycemia, reflective of a T2D phenotype (Reed et al., 2000). Rats were given 10% sucrose water overnight *ad libitum* to prevent potential hypoglycemia that can occur during the first 24–48-h period post STZ because of transient hyperinsulinemia from selective ß-cell destruction (Furman, 2021). For the remainder of the study, STZ-treated T2D rats (*n* = 6–18) were maintained on the high-fat diet, along with HFF control rats not treated with STZ (*n* = 3).

The general glycemic management protocol for diabetic rats (Figure 1B), involved daily blood glucose measurements and a corresponding dose of basal/bolus insulin treatment (insulin glargine; Sanofi-Aventis, Bridgewater, New Jersey; and insulin lispro, Eli Lily, Canada). Insulin dosage (subcutaneous route [SQ]) was titrated based on evening blood glucose measurement as follows: for blood glucose 15.1–20 mmol/L, animals were administered 2 U/day of insulin glargine, animals between 20.1–25 mmol/L received 3 U/day and animals over 25.1 mmol/L were given 4 U/day. This was carried out on all days except on the days after insulin tolerance tests or hypoglycemic challenge. All rats underwent stimulated (i.e., oral glucose tolerance test [OGTT], insulin tolerance test [ITT]) and unstimulated (resting, postabsorptive) assessments (challenges), with and without SSTR2 antagonist pre-treatment dosed 60 min (t = −60) prior to either glucose or insulin administration at t = 0. Each challenge condition was separated by a 1-week washout period.

### 2.2 Blood sampling, blood glucose and plasma hormone measurements

For plasma hormone (glucagon and c-peptide) sampling, EDTA-coated microtubes (Cat # 16.444.100, Sarstedt, Canada) were used to collect ∼200 µL of whole blood from a saphenous vein bleed using a sterile needle (25G). All blood samples were immediately centrifuged at 12000 rpm for 5 min at 4°C to enable separation of plasma from blood cells. Plasma was then removed, and samples were aliquoted and stored at −80°C for subsequent hormone analysis. Blood glucose was measured using glucometers (ContourNext^®^ glucose meter and test strips, Ascensia Diabetes Care, Mississauga Canada) via a tail or saphenous vein bleed using a sterile 30G needle and ∼20 μl of blood. Whole blood from the saphenous vein was also used to assess hemoglobin A1c (HbA1c) level, as an indication of overall glucose control. Commercially available enzyme-linked immunosorbent assay (ELISA) hormone assay kits were used for the determination of plasma c-peptide (Crystal Chem Cat# 90055, RRID:AB_2893130) and glucagon (Mercodia Cat# 10–1271-01, RRID:AB_2737304) levels.

### 2.3 Oral glucose tolerance test

To assess oral glucose tolerance, *n* = 13 rats were randomly selected from the T2D group and *n* = 9 rats from the HFF control group to complete an OGTT. Following a 12-h fast, animals received an oral gavage of reagent-grade D-glucose (BioShop, Burlington, ON, Canada) at a dose of 2 g/kg. Blood glucose concentration was measured at time points of 0, 5, 10, 20, 30, 45, 60, 90, 120 min post glucose gavage. Plasma samples were obtained at t = 0, 30, and 120 min for c-peptide and glucagon analysis.

### 2.4 Insulin tolerance test/hypoglycemic challenge

An insulin tolerance test was conducted to assess insulin resistance/dysglycemia in both T2D and HFF controls. For this, a subset of animals (*n* = 3 each) were food-restricted (15–30 g) during the 24-h period before and administered rapid-acting insulin (NovoRapid, Novo Nordisk, Mississauga, Canada) at 09:00 on the morning of the ITT. HFF controls had lower baseline blood glucose levels compared to animals with T2D and are more sensitive to insulin than the insulin resistant T2D animals. Therefore, due to lower baseline glucose and differences in insulin sensitivity, the HFF animals required a lower dose of insulin to achieve hypoglycemia compared to animals with T2D. The dose of insulin aspart was given subcutaneously at 12 U/kg for the T2D group and 2 U/kg for the HFF control rats, accounting for different baseline glucose levels and expected differences in insulin sensitivities. Blood glucose was monitored via tail nick bleed approximately every 10 min for 180 min. Plasma samples were also collected intermittently for up to 180 min for the glucagon and c-peptide measurements.

Hypoglycemic “challenges” were conducted in a manner similar to the ITT procedure as described above. HFF, NCF, and T2D rats were food-restricted the night before the challenge. Animals were administered (SQ for all) the SSTR2 antagonist PRL-2903 (10 mg/kg, Zucara Therapeutics) or vehicle 1 h prior (t = −60) to insulin dosage (3 U/kg insulin aspart) at t = 0 min. Blood glucose levels were measured at t = −60, −30, 0 and intermittently until either t = 100 or t = 120 depending on the sub-study. Blood samples were collected to measure plasma glucagon and c-peptide levels at t = −60, 0, 30, 60, and 90 min. The protocol was then repeated in a second group of T2D rats maintained on insulin glargine for basal insulin therapy treatment and in control rats on a HFF diet, along with 3 mg/kg ZT-01 (Zucara Therapeutics, Toronto Canada) or vehicle, following an insulin “overdose” of 12 U/kg insulin aspart in an attempt to induce iatrogenic hypoglycemia (i.e., a blood glucose <70 mg/dL). Portal plasma samples and liver tissue were obtained during animal harvest under anesthesia using isoflorane at the end of the ITT.

### 2.5 Pharmacodynamics

Assessments of hormone (glucagon, c-peptide) and blood glucose responses to drug administration under basal (unstimulated) conditions of hyperglycemia and euglycemia were conducted in a subset of T2D and control rats (*n* = 3–4 rats per group). To assess each condition, rats were food-restricted the night before (∼15 g of chow, starting at 17:00) and administered 3 mg/kg of ZT-01, or vehicle on the morning of the test at 09:00. Blood glucose levels were recorded pre and post-treatment (every 30 min for 4 hours). A final blood sample was collected 24-h post-dose. Plasma samples to assess glucagon and c-peptide concentrations were obtained at t = 30, 60, 120, and 240 min.

### 2.6 Statistical analysis

Statistical analysis was carried out using an unpaired *t*-test with Welsh’s correction or a one/two/three-way ANOVA as applicable with a Tukey or Bonferroni test as specified. All data were summarized as mean ± SD. Statistical significance was indicated as applicable and described in further detail in the corresponding figure legends.

## 3 Results

### 3.1 Characteristics of the T2D rodent model

Following STZ treatment, fed blood glucose levels, as measured at 09:00, were 22.7 ± 3.8 mmol/L in the T2D rats (Figure 1B). Mean levels remained within a range of 20–22 mmol/L (mean: 21.8 ± 4.7 mmol/L) over the course of the study. HFF control animals had a mean fed blood glucose concentration of 6.5 ± 0.7 mmol/L (Figure 1B) while normal chow-fed (NCF) control rats exhibited a fed glucose of 5.9 ± 0.6 mmol/L (data not shown). Animals with T2D had HbA1c 8.6% ± 0.6% (*n* = 6) (Figure 1C), which was significantly higher than HFF controls (5.5% ± 0.5%, *n* = 3), or NCF control rats (4.9% ± 0.9%, *n* = 19). Additionally, animals with T2D were found to have on gross examination, higher levels of ectopic fat (liver, muscle, visceral fat) as depicted in Supplementary Figure S1. These data collectively (daily glucose levels, glucose tolerance, baseline hormone and glucose measurements described in the upcoming sections) demonstrated that all animals exhibited the T2D phenotype. No animals were excluded based on blood glucose level or health status; however, the number of rats was in each group was not balanced for research priority, efficiency and cost reasons.

### 3.2 Blood glucose and hormone response under conditions of glucose (OGTT) and insulin tolerance (ITT) without SSTR2 antagonist

#### 3.2.1 Glucose Tolerance

**FIGURE 2:**
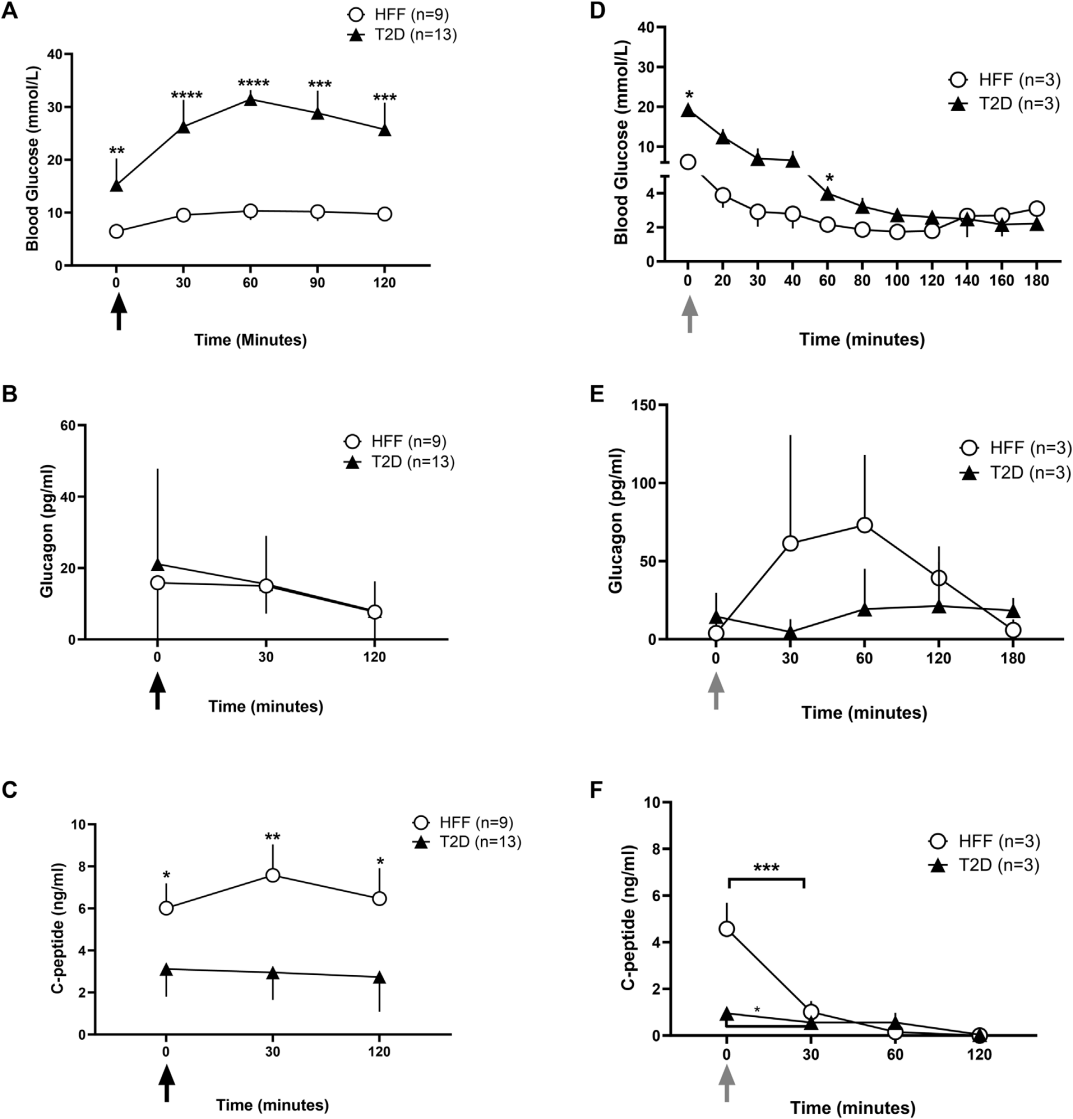
Blood glucose and hormone data under stimulated conditions without SSTR2 antagonist. Whole blood glucose and plasma hormone levels were recorded during stimulated conditions of glucose denoted by the black arrows **(A–C)** and insulin tolerance denoted by the grey arrows **(D–F)**. Blood glucose levels **(A and D)** during an oral glucose tolerance test (OGTT) and insulin tolerance test (ITT) over 120 min post glucose **(A)** or insulin **(B)** administration respectively at t = 0 min were reported for HFF controls and animals with T2D. Hormone analysis for plasma glucagon **(B and E)** and c-peptide **(C and F)** was also conducted at baseline and at regular intervals thereafter for both conditions. Statistical differences indicated are between HFF and T2D groups as *p < 0.05, **p < 0.01, ***p < 0.001, ****p < 0.0001. All data are represented as means ± SD.

On the morning of the OGTT, T2D rats had significantly (*p* = 0.009) higher fasting blood glucose concentrations (15.4 ± 5.1 mmol/L) compared to the HFF controls (HFF: 6.4 ± 0.6 mmol/L) (Figure 2A). Within 30 min of oral glucose administration, blood glucose levels rose markedly in both groups (Figure 2A), increasing by 10.7 ± 2.9 mmol/L in the T2D rats within the first 30 min (*p* < 0.001), and more modestly in the HFF controls (Δ: 3.0 ± 1.5 mmol/L). The magnitude of change from baseline to peak glucose level was higher in the T2D group (Δ: 16 ± 5.1 mmol/L) compared to the HFF control group (Δ: 3.9 ± 1.8 mmol/L) (*p* < 0.001). At the completion of the OGTT (t = 120 min), glucose levels in the HFF group remained somewhat elevated compared to baseline values (∆: 3.3 ± 0.9 mmol/L), suggestive of insulin resistance but not T2D *per se*, while the T2D rats had sustained hyperglycemia (Δ: 10.2 ± 5.8 mmol/L, *p* < 0.01 vs. HFF controls at the same time point).

Baseline C-peptide levels were ∼50% lower in the T2D rats than in the HFF controls (3.1 ± 1.3 vs. 6.1 ± 1.2 ng/mL, *p* < 0.05) (Figure 2C), and remained largely unresponsive to the OGTT in the T2D rats. The T2D rats also tended to have higher plasma glucagon levels at baseline than HFF controls, although this difference was not statistically significant (21.1 ± 26.7 vs. 15.8 ± 17.1 pg/mL, *p* = 0.05). In both groups, glucagon levels declined over the duration of the OGTT, reaching ∼15 pg/mL at t = 30 min and terminating at ∼ 7.7 pg/mL in both groups, with no obvious group-related differences (Figure 2B).

#### 3.2.2 Insulin tolerance/hypoglycemic challenge

An ITT was performed on a subset of animals (*n* = 3 each) in the HFF and T2D groups to first examine potential group differences in insulin sensitivity and to help optimize the hypoglycemia induction model before testing SSTR2 antagonist treatment. During the ITT, T2D rats had a higher baseline blood glucose level vs. HFF controls (19.3 ± 1.0 mmol/L vs. 6.1 ± 0.6 mmol/L, *p* < 0.001) and values remained elevated in the T2D group after insulin bolus (Figure 2D). A difference in the declining trajectory between the two groups was noted at t = 140 min (Figure 2D), in which the T2D group continued to decline, while the HFF group had already reached a nadir (2.7 ± 1.3 mmol/L).

During the ITT, glucagon levels tended to be higher in the HFF rats as compared to the T2D rats, however, these differences were not statistically different (Figure 2E). C-peptide levels dropped in each group during the ITT, however, the HFF animals had much higher baseline c-peptide values than the T2D group, and thus the drop (change from baseline) was more pronounced in the HFF group (Figure 2F).

### 3.3 SSTR2 antagonist treatment under basal conditions

**FIGURE 3:**
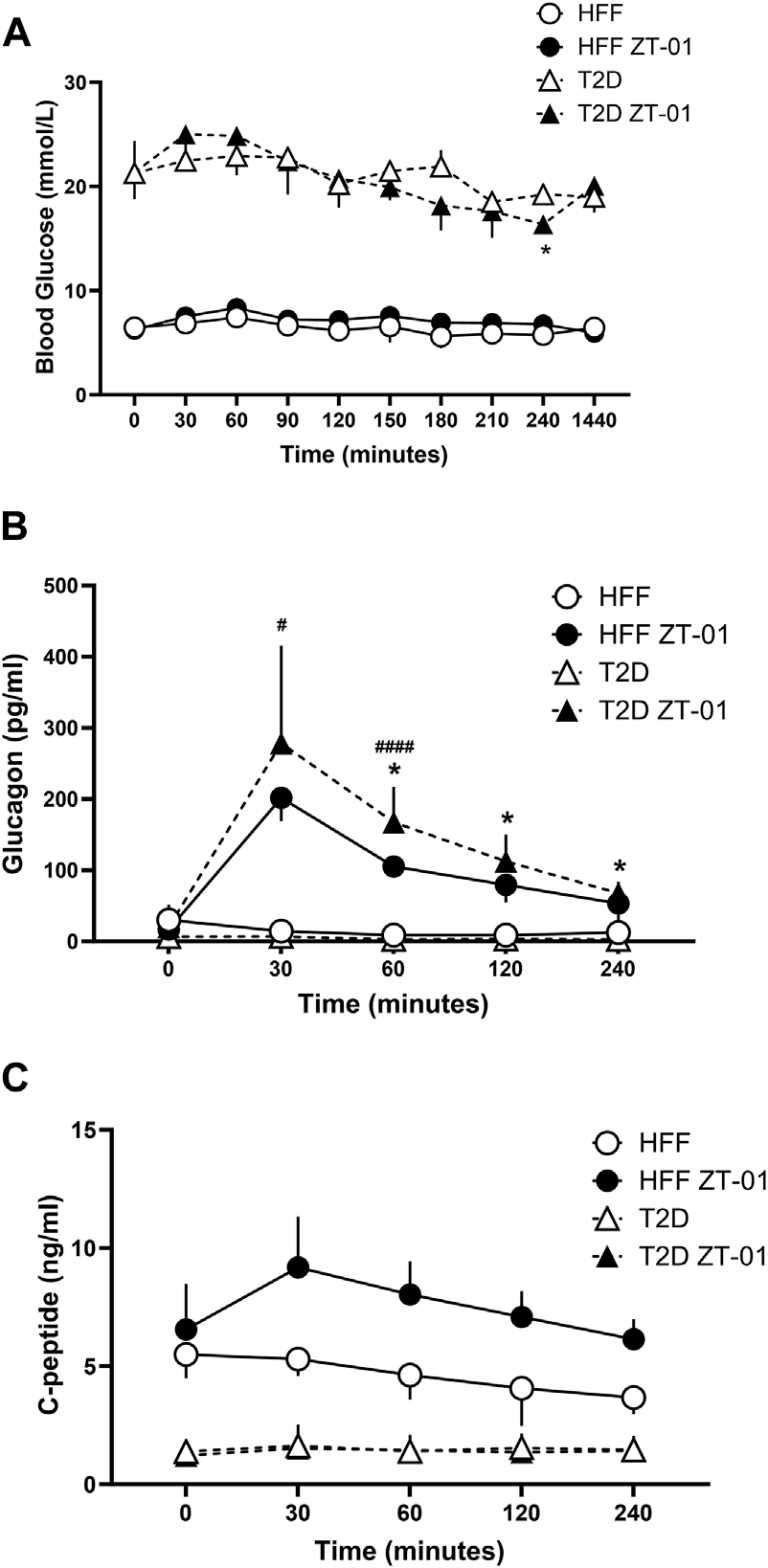
Hormone and glucose responses to SSTR2 antagonist administration in hyperglycemia and euglycemia in T2D and HFF controls. Animals were administered ZT-01 (SSTR2 antagonist) at t = 0 and blood glucose **(A)** and hormone levels for glucagon **(B)** and c-peptide **(C)** were recorded at baseline and at periodic intervals thereafter. Statistical differences indicated between drug and vehicle-treated groups are indicated as *p < 0.05 for T2D, while differences between HFF treatment groups are indicated as ^#^ *p* < 0.05 and ^####^ *p* < 0.0001. All data is represented as mean ± SD.

The effect of the SSTR2 antagonist ZT-01, was assessed under basal conditions of hyperglycemia (T2D animals only) and euglycemia (HFF controls only) in the absence of any manipulations to glucose levels such as those carried out for the OGTT and ITT. Blood glucose responses (Figure 3A) to SSTR2 antagonist administration (ZT-01) were not significantly different between T2D and HFF controls at any timepoint, except for the t = 240 min time point when animals treated with ZT-01 (3 mg/kg) displayed significantly lower blood glucose than the T2D vehicle group (16.4 ± 0.8 vs. 19.2 ± 0.8 mmol/L, *p* < 0.05). Although blood glucose at the other time points was not significantly different between treatments, there was a trend toward higher blood glucose levels immediately (or at least within 60 min) post ZT-01 administration, followed by a steady decline in glucose values after –120 min, to a level similar to that observed in the vehicle controls. Beyond this time point, ZT-01-treated animals had lower glucose compared to vehicle controls with the lowest levels noted at t = 240 min. Glucose levels between drug and vehicle-treated groups were comparable at 24-h post-dosing.

Plasma glucagon responses (Figure 3B) increased in both ZT-01 treated groups (T2D and HFF controls) peaking at t = 30 min, with significantly higher levels (*p* < 0.05) observed in the HFF-ZT-01 treatment group (201.7 ± 32.4 pg/mL) compared to HFF vehicle group (14.7 ± 14.7 pg/mL). Although glucagon started to decline after 30 min (t = −30) post SSTR2 antagonism treatment, plasma values were still significantly higher in the respective vehicle-treated control groups (i.e., up to 60 min post-treatment for the HFF group [*p* < 0.001] and for all time points after 30 min for the T2D groups [*p* < 0.05 for all time points]). C-peptide levels of both ZT-01-treated groups (i.e., T2D or HFF) were not significantly different from their vehicle-treated controls at any time point (Figure 3C). Nonetheless while the C-peptide response in the T2D group remained, with ZT-01 treatment the HFF-ZT-01 rats had higher mean c-peptide levels with SSTR2 antagonist administration starting at t = 30 min (5.3 ± 0.7 vs. 9.2 ± 2.2 ng/mL) and at the t = 240 min time point (3.7 ± 0.7 vs. 6.1 ± 0.8 ng/mL), but the difference was not statistically significant (*p* = 0.06).

### 3.4 SSTR2 antagonist treatment under insulin-induced hypoglycemic challenge conditions

Once model verification assessments were completed and basal hormone and glucose responses determined, two SSTR2 antagonist compounds (PRL-2903 and ZT-01) were tested for hypoglycemia prevention in T2D rats and controls in two experiments. In the first experiment, PRL-2903 (10 mg/kg, intraperitoneal [IP]) was compared to a T2D control group and both T2D groups were compared to non-diabetic HFF controls. In the second experiment, ZT-01 (3 mg/kg, SQ) was compared to a T2D control group. Furthermore, the first hypoglycemic challenges (PRL-2903 treated) were accomplished with 3 U/kg bolus insulin (SQ), and the second set of challenges (ZT-01) used a 12 U/kg insulin dose (SQ).

#### 3.4.1 Effect of PRL-2903 treatment

**FIGURE 4:**
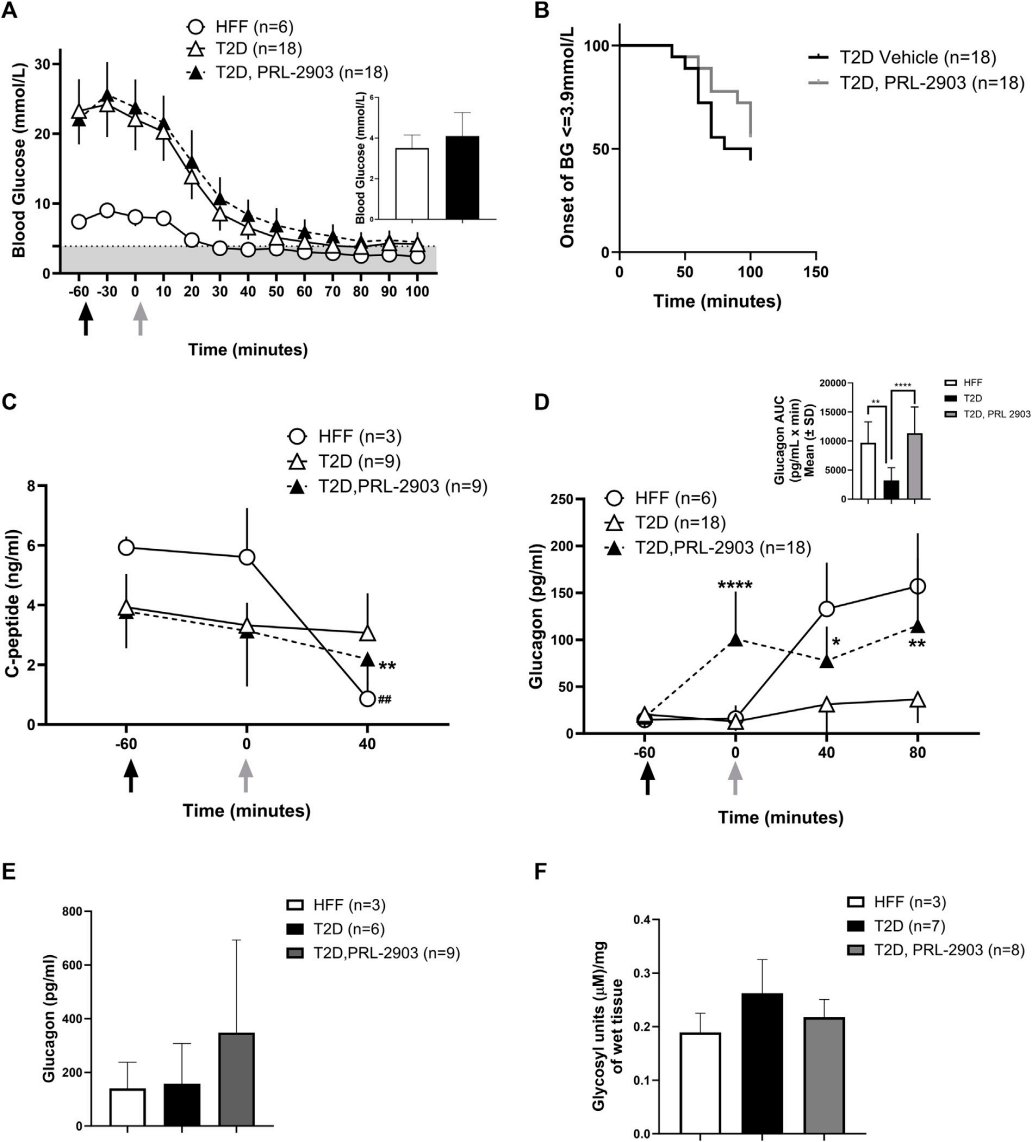
Blood glucose response to hypoglycemic challenges with SSTR2 antagonist PRL-2903: Insulin-induced hypoglycemic challenges were conducted in a crossover design (results represented as combined data). Black arrows indicate the timing of PRL-2903 administration while grey arrows are indicative of insulin administration. Blood glucose response at baseline (t = −60), t = −30, t = 0 (insulin administration) and every 15 min thereafter is illustrated in **(A)**. The grey-shaded region denotes the hypoglycemic zone. A significant interaction effect of time and treatment was observed (*p* < 0.001). Additional analysis on the rate of hypoglycemia **(B)** is also provided. Hormone responses for c-peptide **(C)** and glucagon **(D)** are also reported with additional representation of glucagon AUC (D′). Portal glucagon levels are reported in **(E)** and liver glycogen levels in **(F)**. Statistical significance is indicated as *p < 0.05, **p < 0.01 for T2D-SSTR2 antagonist vs T2D and ^##^ *p* < 0.01 for HFF. All data are presented as means ± SD.

As expected, rats with T2D had a significantly higher (*p* < 0.05) baseline glucose level than the HFF controls (Figure 4A). The HFF control group reached hypoglycemia much faster than the T2D rats following bolus insulin administration to induce hypoglycemia in both the vehicle- and SSTR2 antagonist-treated (PRL-2903) conditions. Within the T2D group, blood glucose levels were higher with PRL-2903 treatment, as compared to vehicle treatment (main effect, *p* < 0.05), but no treatment by time interaction was found. Although the difference was not significant, the T2D-SSTR2 antagonist-treated animals had a higher nadir glucose (4.1 ± 1.2 mmol/L), slightly above the hypoglycemia threshold of 3.9 mmol/L, as compared to the T2D vehicle group where the average was below the hypoglycemic threshold (3.5 ± 0.6 mmol/L) (*p* = 0.07) (Figure 4A inset). Hypoglycemia did not occur in all animals during the ITT and fewer T2D rats developed hypoglycemia with PRL-2903 treatment (44%) than with vehicle treatment (66%) (Figure 4B).

C-peptide levels in HFF control and T2D-PRL-2903 treated animals during hypoglycemia (Figure 4C) were significantly lower at t = 40 min as compared to baseline (HFF: 5.9 ± 0.4 vs. 0.9 ± 0.6 ng/mL, T2D-PRL-2903: 3.8 ± 1.2 vs. 2.2 ± 0.9 ng/mL, *p* < 0.01 for both comparisons). However, no decline was noted in the T2D vehicle group (3.9 ± 1.1 vs. 3 ± 3.3 ng/mL) under hypoglycemic conditions. PRL-2903-treated T2D rats had similar C-peptide levels as the vehicle—treated T2D rats at baseline, with values dropping slightly but significantly more in the PRL-2903-treated T2D group (*p* < 0.001).

Plasma glucagon levels (Figure 4D) were markedly higher in T2D-PRL-2903-treated animals, within an hour of dosing (which was at t = 0 min), as compared to T2D vehicle controls (100.9 ± 50.6 pg/mL vs. 12.8 ± 9.9 pg/mL, *p* < 0.0001) and glucagon values remained higher throughout the duration of the hypoglycemic challenge. In the T2D-SSTR2 antagonist group, glucagon levels at all later time points were significantly higher than at baseline (i.e., t = −60). Glucagon area under the curve (AUC) response to the hypoglycemic challenge, was significantly higher in the T2D-SSTR2 antagonist-treated group as compared to the T2D vehicle group (11336 ± 4524 vs. 3239 ± 2373, *p* < 0.05), with AUC values in the drug-treated group approaching those observed in the HFF controls (9704 ± 3579) (Figure 4D inset).

Portal plasma levels of glucagon (Figure 4E) were measured at ∼100 min after insulin administration and tended to be higher in the PRL-2903-treated T2D rats (239 ± 344 pg/mL) as compared to the control T2D rats (157 ± 151 pg/mL) or HFF rats (140 ± 97 pg/mL), although the differences were not statistically different and results were highly variable (*p* = 0.32). Liver glycogen concentration also tended to be lower following PRL-2903 treatment, compared with vehicle-treated controls, although the difference was also not statistically significant (*p* = 0.08, Figure 4F). Liver lobes from rats harvested after hypoglycemic challenges had the appearance of steatosis in HFF rats (both HFF controls and T2D rats), whereas liver from NCF animals had a normal appearance (Supplementary Figure S1).

#### 3.4.2 Glycemic and hormonal effects of ZT-01 treatment

**FIGURE 5:**
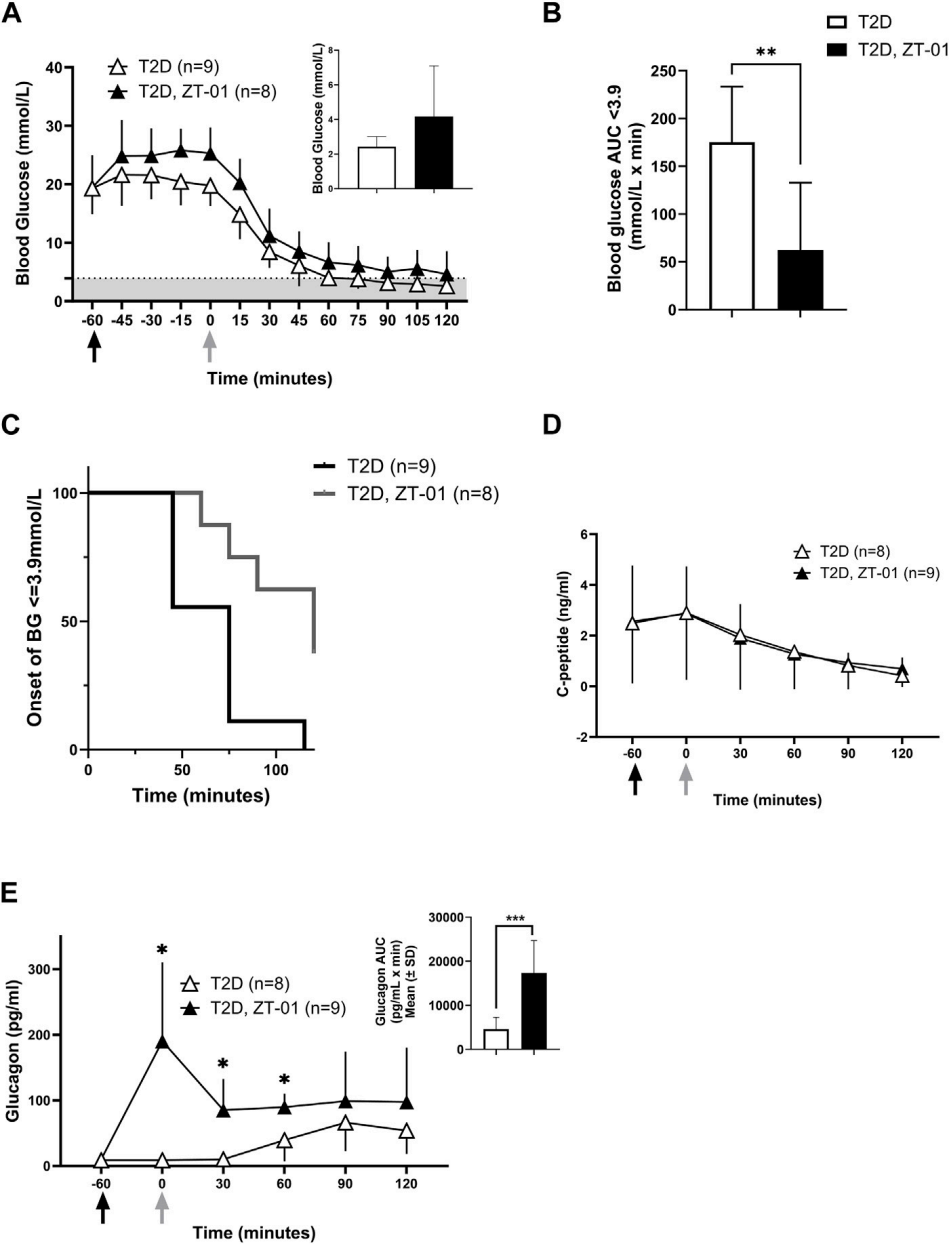
Efficacy of SSTR2 antagonist ZT-01 for hypoglycemia prevention: Insulin-induced hypoglycemic challenges were conducted as described above in a single hypoglycemic challenge. Black arrows indicate the timing of ZT-01 administration while grey arrows are indicative of insulin administration. BG response at baseline (t = −60), t = −30, t = 0 (insulin administration) and every 15 min thereafter is illustrated in **(A)** along with the nadir glucose in each group (A inset). The grey-shaded region denotes the hypoglycemic zone. Additional analysis on time spent in hypoglycemia **(B)** and rate of hypoglycemia **(C)** is also provided. Hormone responses for c-peptide **(D)** and glucagon **(E)** are also recorded with additional representation of glucagon AUC (E inset). A significant interaction effect of time and treatment was observed for glucagon responses (*p* < 0.01). Statistical significance is indicated as *p < 0.05, **p < 0.01 for T2D vs. T2D-SSTR2 antagonist. All data are presented as means ± SD.

Blood glucose levels were monitored as described above during an insulin-induced hypoglycemic challenge in T2D rats with the increased insulin dosage of 12 U/kg (Figure 5A). In the 60 min after drug dosing and prior to insulin administration, glucose appeared to rise more with ZT-01 as compared to the vehicle-treated group, but this difference was not statistically significant at t = 0 vs. t = −60 (adjusted *p* = 0.9 for Bonferroni *post hoc* test at t = 0). The insulin challenge dose was 12 U/kg, which was higher than the 3 U/kg dose used in the PRL-2903 experiment, selected with the intent to induce level 2 hypoglycemia (<3.0 mmol/L) in the control group, whereas level 1 hypoglycemia (3.5 mmol/L blood glucose) was achieved in the PRL-2903 study. Despite a more intense hypoglycemic stimulus, ZT-01-treated T2D rats had a decreased incidence of hypoglycemia relative to vehicle-treated controls. Overall, 37.5% of ZT-01 treated rats maintained blood glucose levels >3.9 mmol/L while all control rats became hypoglycemic (Figure 5C). Protection from hypoglycemia was also reflected in the significantly longer time to hypoglycemia onset in the SSTR2 antagonist-treated group for animals that did develop hypoglycemia (66.1 ± 23.7 vs. 103.1 ± 24.6 min, *p* < 0.01) and the extent of hypoglycemia (as assessed by AUC analysis for blood glucose <3.9 mmol/L) was significantly greater in the T2D vehicle-treated group compared to the ZT-01-treated group (*p* = 0.003) (Figure 5B). Furthermore, glucose nadir and terminal glucose levels were also lower in the T2D-ZT-01 treated animals (Figure 5A inset). No significant differences in c-peptide responses were observed with ZT-01 treatment compared to controls (Figure 5D). Plasma glucagon increased within the first hour following ZT-01 treatment compared to controls, and remained elevated until 1 h after insulin bolus administration for hypoglycemia induction (2 h after ZT-01 dosing) (Figure 5E). Total glucagon exposure (as measured by AUC) was also significantly higher following ZT-01 treatment (*p* = 0.0006) (Figure 5E inset).

**FIGURE 6:**
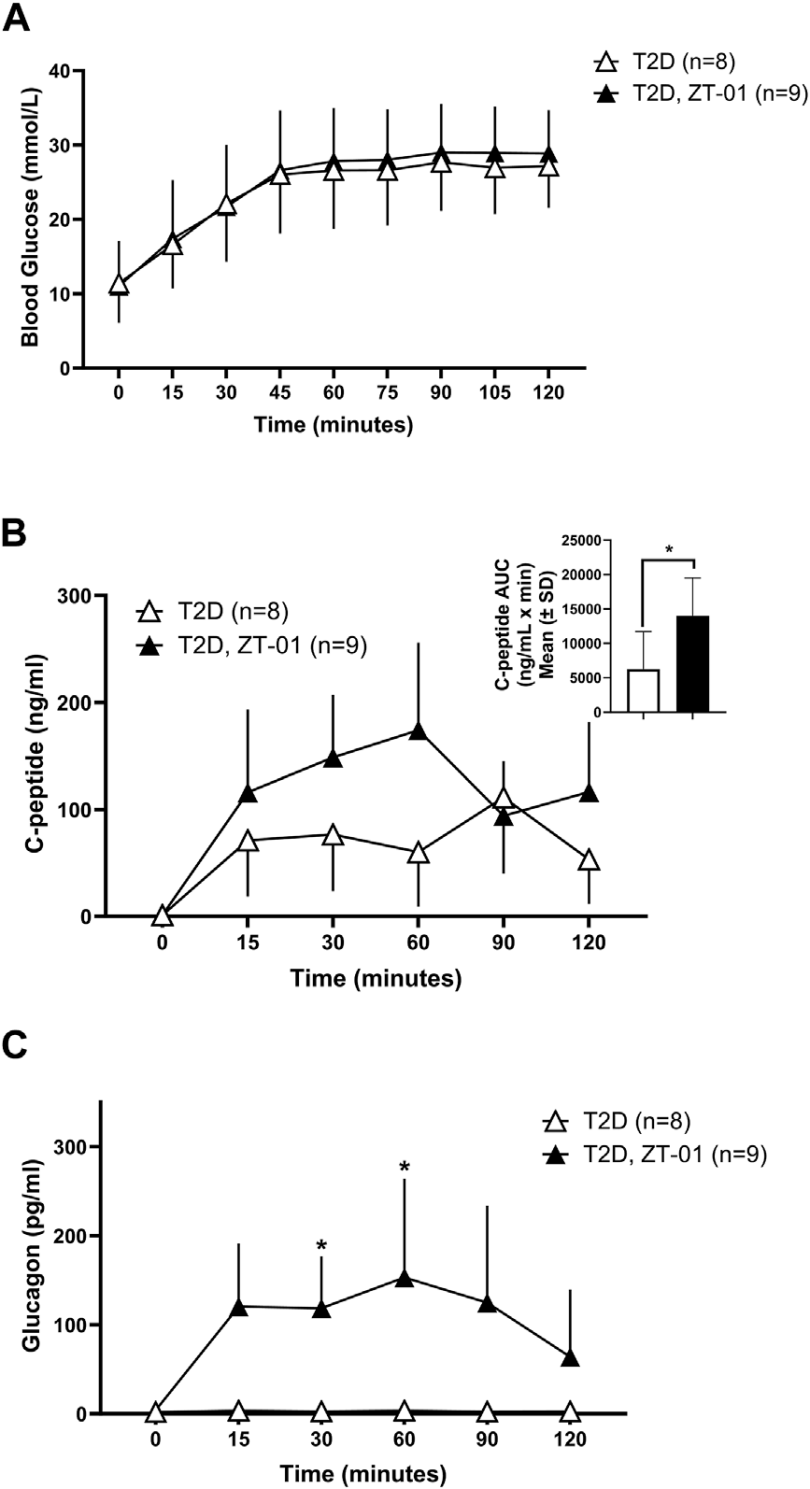
Blood glucose and hormone response to SSTR2 antagonist (ZT-01) under stimulated conditions of OGTT: Blood glucose **(A)** was measured on simultaneous administration of both ZT-01 and glucose at t = 0 and observed until t = 120. Plasma c-peptide **(B)** and glucagon **(C)** responses were also noted over the same time period with additional representation of c-peptide AUC (B inset). A significant interaction effect of time and treatment was observed for glucagon responses (*p* < 0.01). Statistical significance is indicated as *p < 0.05. All data are presented as means ± SD.

ZT-01 treatment under stimulated conditions involving oral glucose administration was also evaluated. Oral glucose administration resulted in a similar rise in blood glucose levels in the ZT-01 treated T2D rats (Figure 6A), as previously observed in the absence of SSTR2 antagonist treatment (Figure 2A). C-peptide levels also increased from baseline by 116% ± 77% in SSTR2 antagonist-treated animals compared to 28.9% ± 70% in T2D controls (Figure 6B). Glucagon rose within 15 min of ZT-01 treatment and glucose administration (120 ± 70 vs. 4.8 ± 3, *p* = 0.006) and remained higher than controls for the duration of the OGTT (Figure 6C).

## 4 Discussion

Prevention of hypoglycemia remains an unmet need for those receiving insulin therapy. The physiologic mechanisms for hypoglycemia development in those on exogenous insulin include the profound glucose-lowering effect of insulin, along with the probability of impaired glucose counterregulation caused by a blunting of several counterregulatory hormones including the catecholamines, cortisol, growth hormone and, in particular, glucagon (Verhulst et al., 2022). Emerging evidence suggests that the blunting of glucagon secretion is related to elevations in SST secretion in sub-optimally controlled diabetes (Gaisano et al., 2012; Rorsman and Huising, 2018) and that the inhibition of SSTR2 can help restore the glucagon response to hypoglycemia, at least in animal models of T1D (Yue et al., 2012; 2013; Karimian et al., 2013). The primary purpose of this study was to test the efficacy of two SSTR2 antagonists in a rodent model of T2D to see if treatment could enhance the endogenous glucagon counterregulatory response and prevent hypoglycemia during an insulin-induced hypoglycemic challenge. Our secondary goal was to assess the potential impact of SSTR2 antagonist treatment on oral glucose tolerance.

With these objectives in mind, we successfully developed a male rodent model of late-stage T2D characterized by significantly higher HbA1c, insulin resistance and defective glucagon counterregulation to insulin-induced hypoglycemia compared to HFF and NCF controls. Consistent with literature findings, c-peptide levels in this rodent model of T2D were lower than those observed with high-fat feeding alone but higher than levels found in rodent models of autoimmune T1D (Karimian et al., 2013). Additionally, decreased and subsequently unresponsive levels of c-peptide noted during the OGTT are reflective of decreased insulin secretion, also noted in humans with T2D, which is accompanied by reduced whole-body insulin sensitivity (Clark et al., 2001).

We hypothesized that treatment with an SSTR2 antagonist (PRL-2903), prior to insulin-induced hypoglycemia, would increase the glucagon response to iatrogenic (i.e., insulin-induced) hypoglycemia. This was confirmed in this study. A glucagon response to hypoglycemia was observed in HFF control animals, but the response was largely absent in the T2D controls. However, when treated with PRL-2903, T2D rats had an increased glucagon response, achieving levels close to the HFF control response (Figure 4D). A similar effect of increased glucagon relative to controls in T2D rats was observed on treatment with ZT-01, although the effect with ZT-01 was more pronounced than with PRL-2903 (Figure 5E). Furthermore, the effect of SSTR2 antagonist on glucagon response was also observed under euglycemic/hyperglycemic conditions (Figure 3), in which glucagon is not normally stimulated, and during stimulated hyperglycemic conditions (Figure 6). Collectively these responses in the SSTR2 antagonist-treated animal groups resulted in glucagon levels greater than in T2D controls and similar to those observed in HFF control response to hypoglycemia. The effect on preventing hypoglycemia onset was also more pronounced with 3 mg/kg ZT-01 than 10 mg/kg PRL-2903, indicating ZT-01 was more potent in this model. ZT-01 treatment prevented hypoglycemia in 37% of rats who were administered bolus insulin to induce mild-to-moderate hypoglycemia (Figure 5C). The attenuating effects of ZT-01 on iatrogenic hypoglycemia have also been reported earlier in a rat model of T1D (Farhat et al., 2022).

Additionally, a more robust hypoglycemia challenge was used to assess ZT-01 (12 U/kg insulin, achieving blood glucose in controls <3.0 mmol/L), compared to PRL-2903 (3 U/kg, achieving glucose values of 3.5 mmol/L in controls). After the initial demonstration of SSTR2 antagonist efficacy using PRL-2903, a lower glucose nadir was targeted, to ensure that a high proportion of control rats would experience hypoglycemia. The lower glucose target was also selected to provide the best opportunity for rats to have a stimulated counterregulatory response, ensuring the control rats would have their maximal, albeit impaired response, and thus providing the most robust comparison for the ZT-01 treated group. Overall, therefore, both antagonists resulted in higher glucagon concentrations and higher glucose nadirs that were above the hypoglycemic threshold following insulin bolus overdose.

Nocturnal hypoglycemia is a major concern for most individuals living with diabetes who are on insulin therapy, (Ratzki-Leewing et al., 2018; Siamashvili et al., 2021). It is a much feared complication in diabetes because nocturnal hypoglycemia may not produce sufficient symptoms to signal an individual to self-treat and because it can be fatal (Jones et al., 2022). Administration of an SSTR2 antagonist at bedtime could be one potential new therapeutic approach to reduce its occurrence. The timing of dosing requires consideration. At present the ideal timing or dose level for regular use of SSTR2 antagonists for the prevention of hypoglycemia remains unclear. The 60-min time point prior to hypoglycemia for SSTR2 antagonist drug dosing was selected in these studies based on the previously reported PK/PD results for PRL-2903 and ZT-01 (Farhat et al., 2022), showing peak plasma drug levels were achieved −2 h after subcutaneous injection, and that glucagon levels increase within 60 min of treatment (as also observed in this study, at 30 min post-dose). Given that glucagon levels rise within an hour after ZT-01 dosing, we also assessed the potential for ZT-01 administration to exacerbate hyperglycemia when glucose levels are stimulated, such as after feeding (which we simulated using an OGTT). ZT-01 was dosed immediately before administering oral glucose, to align the onset of ZT-01-induced glucagon response with the post-absorptive peak glucose level which typically occurs 60 min after administration. However, this did not overtly compromise oral glucose tolerance in this model of T2D. However, both antagonists tested tended to result in a slight rise in glucagon level post-dose. Therefore drug administration might best be performed when glucose levels drop several hours after feeding. On the other hand, ZT-01 appeared to result in an acute increase in c-peptide levels when given prior to the OGTT which could be an indirect effect of increasing glucagon secretion and potentially glycemia. This might suggest that drug dosing prior to meals may also be feasible.

Other labs (Capozzi et al., 2019) have shown that stimulation of β cells via high glucose exposure, along with glucagon treatment, increases insulin secretion up to 5-fold compared to stimulation with high glucose alone. Thus, the increased plasma glucagon response following SSTR2 antagonism combined with the glucose administration during the OGTT may explain the rise in c-peptide levels noted in the ZT-01 group compared to controls (Figure 6). This could be augmented due to a possible incretin effect, such as a rise in glucagon-like peptide secretion (Seino et al., 2010), which has yet to be investigated. Gut-derived incretin hormones have been shown to specifically contribute to increased post-prandial insulin levels, particularly GLP-1 (Holst, 2019). Recent findings have described a role for both SSTR5 antagonism and to a lesser extent, SSTR2 antagonism in mediating reduction in blood glucose through gut-dependent GLP-1 stimulation of insulin release (Jepsen et al., 2021). The potential for an effect of ZT-01 on the incretin hormones and a resultant effect on insulin and glucagon secretion has so far not been investigated, and further investigation to elucidate this mechanism is an area of focus in our lab and will be addressed in upcoming research studies.

In the current study, the increased time before hypoglycemia onset, and the decreased incidence of hypoglycemia following SSTR2 antagonist treatment (PRL-2903, Figure 4B) was accompanied by a corresponding increase in plasma glucagon level and a decrease in liver glycogen content (Figure 4F, *p* = 0.08). Liver glycogen content is normalized, or even elevated, with exogenous insulin treatment in T1D (Chatila and West, 1996; Tsujimoto et al., 2006; Julián et al., 2015) and this phenomenon appears to be reflected in our model of T2D when compared to HFF controls (Figure 4E). The elevation in hepatic glycogen content in the rats with T2D may be the result of high circulating blood glucose levels combined with hyperinsulinemia with insulin therapy (Chatila and West, 1996; Julián et al., 2015). Apart from inhibiting energy storage and utilization processes, the primary action of glucagon is in stimulating hepatic glycogenolysis and gluconeogenesis (Barthel and Schmoll, 2003; Ramnanan et al., 2011; Trefts et al., 2017). Previous experiments in our lab assessing hypoglycemia prevention using an SSTR2 antagonist in a rodent model of recurrent hypoglycemia have also demonstrated a similar decrease in liver glycogen content with drug treatment (Hoffman et al., 2021). These findings support the notion that SSTR2 antagonism acts directly as an α-cell glucagon stimulant into the hepatic portal vein circulation, which in turn results in rapid hepatic glycogen release. This approach may be superior to the peripheral administration of much larger doses of glucagon to achieve the same level of hepatic glucagon exposure, which can cause adverse side effects such as nausea and vomiting (Story and Wilson, 2022).

This study has several strengths and limitations that need to be acknowledged. First, we developed a rodent model of late-stage T2D that involved rigorous model characterization, extensive insulin titration protocols for adequate depth and duration of hypoglycemia and efficacy testing with two SSTR2 antagonist compounds. We performed glucose and hormone characterization with and without SSTR2 antagonist under basal (unstimulated) and hypoglycemia-stimulated conditions in animals with and without T2D. However, in some of these experiments, the number of animals was small (*n* = 3–5), particularly in the normal chow (healthy) rat groups, and thus these data should not be deemed as conclusive. Additionally, only male rats were used in these studies and did not include a female subset of the T2D model. Although sex differences were beyond the scope of this study, additional studies will be conducted as a follow-up to assess any differences in insulin sensitivity and subsequent efficacy of ZT-01 for hypoglycemia prevention in female animals with T2D. Future studies will also assess the effect of chronic drug dosing to determine if drug treatment effects attenuate over time.

In summary, we show here that an SSTR2 antagonist can improve the glucagon counterregulatory response to insulin-induced hypoglycemia and prevent, or at least delay, the onset of hypoglycemia by up to −40 min in a rat model of T2D. Future investigations using this rodent model are being undertaken to examine SSTR2 antagonist effects on glycemia, glucagon and incretin levels in various stimulated and unstimulated conditions using single and repeat dosing paradigms, and to assess if there may be sex differences in the biological response to acute and chronic SSTR2 antagonism.

## Data Availability

The raw data supporting the conclusion of this article will be made available by the authors, without undue reservation.

## Footnotes

1
https://classic.clinicaltrials.gov/ct2/show/NCT05007977?cond=Diabetes&lead=zucara&draw=2&rank=2

2 EASD 2023 abstract 61: Abitbol et al. (2023). ZT-01, a somatostatin receptor 2 antagonist, increases glucagon response to hypoglycemia in a phase 1b glycaemic clamp study in type 1 diabetes.

3
https://classic.clinicaltrials.gov/ct2/show/NCT05762107?cond=Diabetes&lead=zucara&draw=2&rank=1

## Supplementary material

SUPLEMENTARY FIGURE S1 Liver lobes obtained immediately post-harvest at t=100 at the end of the hypoglycemic challenge. Picture denotes increased liver adiposity for animals on high-fat diet and animals with diabetes in comparison to healthy animals on a normal chow diet.
