# Supplementary figures and images for: Evaluating the effectiveness of a novel somatostatin receptor 2 antagonist, ZT-01, for hypoglycemia prevention in a rodent model of type 2 diabetes
Source: Front Pharmacol. 2024 Feb 28;15:1302015. doi: 10.3389/fphar.2024.1302015 (PMC10951717; doi:10.3389/fphar.2024.1302015)

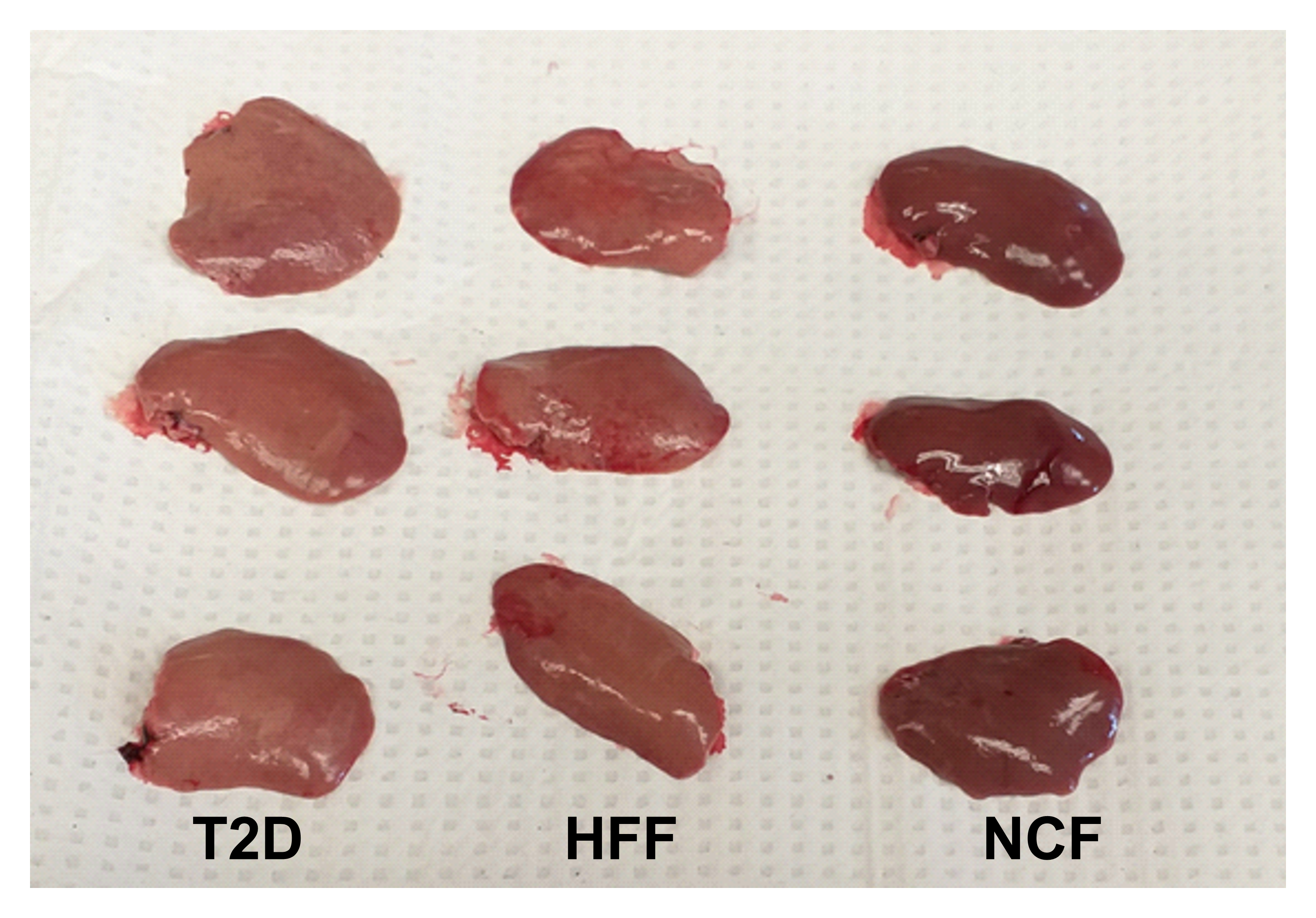

Supplement: Supplementary file 1 [file Image1.TIF]
